# Efficacy and safety of a self-developed home-based enhanced knee flexion exercise program compared with standard supervised physiotherapy to improve mobility and quality of life after total knee arthroplasty: a randomized control study

**DOI:** 10.1186/s13018-021-02516-0

**Published:** 2021-06-14

**Authors:** Tianyang Xu, Dong Yang, Kaiyuan Liu, Qiuming Gao, Hengli Lu, Yue Qiao, Chunyan Zhu, Guodong Li

**Affiliations:** 1 Department of Orthopedics, Shanghai Tenth People’s Hospital, Tongji University School of Medicine, 301 Yanchang Rd, Shanghai, 200072 People’s Republic of China; 2 Tongji University School of Medicine, Shanghai, People’s Republic of China; 3 Department of Operating Room, Shanghai Tenth People’s Hospital, Tongji University School of Medicine, Shanghai, People’s Republic of China

**Keywords:** Home exercise program, Supervised physiotherapy, TKA, Rehabilitation

## Abstract

**Background:**

This randomized controlled study compared standard supervised physiotherapy (SPT) with a self-developed, home-based, enhanced knee flexion exercise program involving a low stool (KFEH) in patients who underwent total knee arthroplasty (TKA).

**Methods:**

Patients were recruited from July 2014 to December 2015 and randomly assigned to one of two groups: KFEH (*n* = 60) and SPT (*n* = 59). Outcomes (joint function) were evaluated according to the Knee Society Score (KSS), visual analog scale (VAS), Western Ontario and McMaster Universities Osteoarthritis Index (WOMAC) score, and range of motion (ROM) assessment at selected time points (preoperatively; 1 week; 1, 3, and 6 months; and 1 year after surgery).

**Results:**

Pain and functional improvement were observed in both groups. Non-inferiority of KFEH was evident 12 months postoperatively; however, patients in the KFEH group exhibited better ROM at 1 month (*P* < 0.01). Absolute WOMAC and KSS scores were slightly better in the KFEH group, although the difference was not statistically significant. There was no difference in VAS scores and complication rates between the two groups. Additionally, the home program would save patient time and decrease the economic burden associated with in-hospital SPT.

**Conclusion:**

Considering rehabilitation and economic efficiency as well as the COVID pandemic, a home-based enhanced knee flexion exercise program for TKA rehabilitation is recommended.

**Supplementary Information:**

The online version contains supplementary material available at 10.1186/s13018-021-02516-0.

## Background

Total knee arthroplasty (TKA) is a highly successful and widely accepted surgical technique for osteoarthritis (OA) [1]. After the surgical procedure, early and sufficient rehabilitation, including physical therapy, significantly contributes to restoring function and range of motion (ROM) in the knee [2]. Physical therapist-supervised programs are a commonly used standard for functional rehabilitation for patients who undergo TKA. Typically, such programs are supervised by a physical therapist during the hospital stay and in an outpatient facility for approximately 10–20 weeks postoperatively [3]. Clearly, such a standard program requires professional and licensed therapists and the appropriate equipment and should be performed in the hospital or an accredited outpatient facility. As such, these post-hospitalization programs require more outpatient facility visits (2–3 times/week) and related costs [4]. Time and economic burdens have, therefore, limited the use of these programs.

Numerous efforts have been focused on cost-effectiveness analysis and controlling the costs of therapist-guided rehabilitation programs. There has also been an urgent pursuit of low-cost and practicable alternatives, such as self-administered home programs [5–8]. Besides, staying at home is helpful to reduce transmission of the coronavirus under the COVID-19 pandemic scenario. Compared with physical therapist-supervised programs, these self-administered home programs are more likely to be accepted by patients without temporal and space limitations as well as any additional costs. In addition, system review demonstrated that home-based protocols did not show an overall significant difference in the outcomes achieved with the supervised one within the studies reviewed [9]. Home exercise programs usually include a telecare component and a standardized regimen. One of the disadvantages of home-based programs is that they are usually not based on standardized protocols, which lead to wide variations in rehabilitation-promoting effects. Additionally, it cannot be guaranteed that the patients themselves will complete―or at least are compliant with―home-based exercise programs. According to our experience, some patients in home-based exercise programs usually exhibit poor knee flexion, which influences body function and satisfaction with surgery. Home tele-rehabilitation guidance for patients undergoing TKA has been developed from standardized home-based exercise programs in recent years due to advances in technology [9, 10]. However, due to economic conditions and the medical input of local districts, telecare is not widely used. Therefore, a current challenge in this field is to introduce standardized self-administered home programs that can reduce knee stiffness and enhance knee flexion.

In the present prospective, randomized, positive-controlled clinical trial, we introduced a self-developed, home-based, enhanced knee flexion exercise (KFEH) program, which involved the use of a low stool (30–40 cm in height) and an exercycle, for patients with OA who underwent TKA. The overall rehabilitation-promoting effect of this program was compared with standard supervised physiotherapy (SPT). We hypothesized that postoperative rehabilitation using KFEH is as at least as efficient as that of the SPT.

## Methods

### Study design

This prospective, randomized, positive-controlled clinical trial was approved by the Institutional Medical Ethics Committee of the authors’ hospital (approval number: SHSY-IEC-KY-4.0/16-19/01) and was conducted in accordance with approved guidelines. The trial was registered with the Chinese Clinical Trial Registry (http://www.chictr.org.cn/, ChiCTR-IOR-17011264) and informed written consent was obtained from all participants.

### TKA procedure

TKA was performed through a midline vertical incision and medial parapatellar approach by two highly experienced chief surgeons. Posteriorcruciate stabilizing prostheses were implanted in all patients without patellar resurfacing, and local infiltration analgesia was applied around the surgical fields as previously described [5, 6]. Incision closure and wound care were performed as per standard protocol in all patients. After surgery, all patients were administered 50 mg flurbiprofen axetil injection twice daily for 1 week to relieve pain, 10 mg rivaroxaban for 2 weeks to prevent deep vein thrombosis, and 1.5 g cefuroxime twice daily for 3 days to prevent infection. Patients were administered pethidine (50 mg), if necessary, for intolerable pain. Each patient stayed in hospital for 7 days and underwent assisted, supervised physical therapy on postoperative day 1.

### Participants

According to a previous study and projected drop-out rate of 15%, 110 patients were required [11]. To compensate for dropouts and deviation from data normality, 60 patients with OA, who were scheduled to undergo TKA between July 2014 and December 2015, were enrolled in the study. Patients < 40 or > 80 years of age, those undergoing revision surgery, those experiencing lower limb ischemia, acute trauma or fracture, or systemic or neuromuscular diseases and those with intellectual disorders were excluded.

### Randomization and treatment allocation

**Fig. 1:**
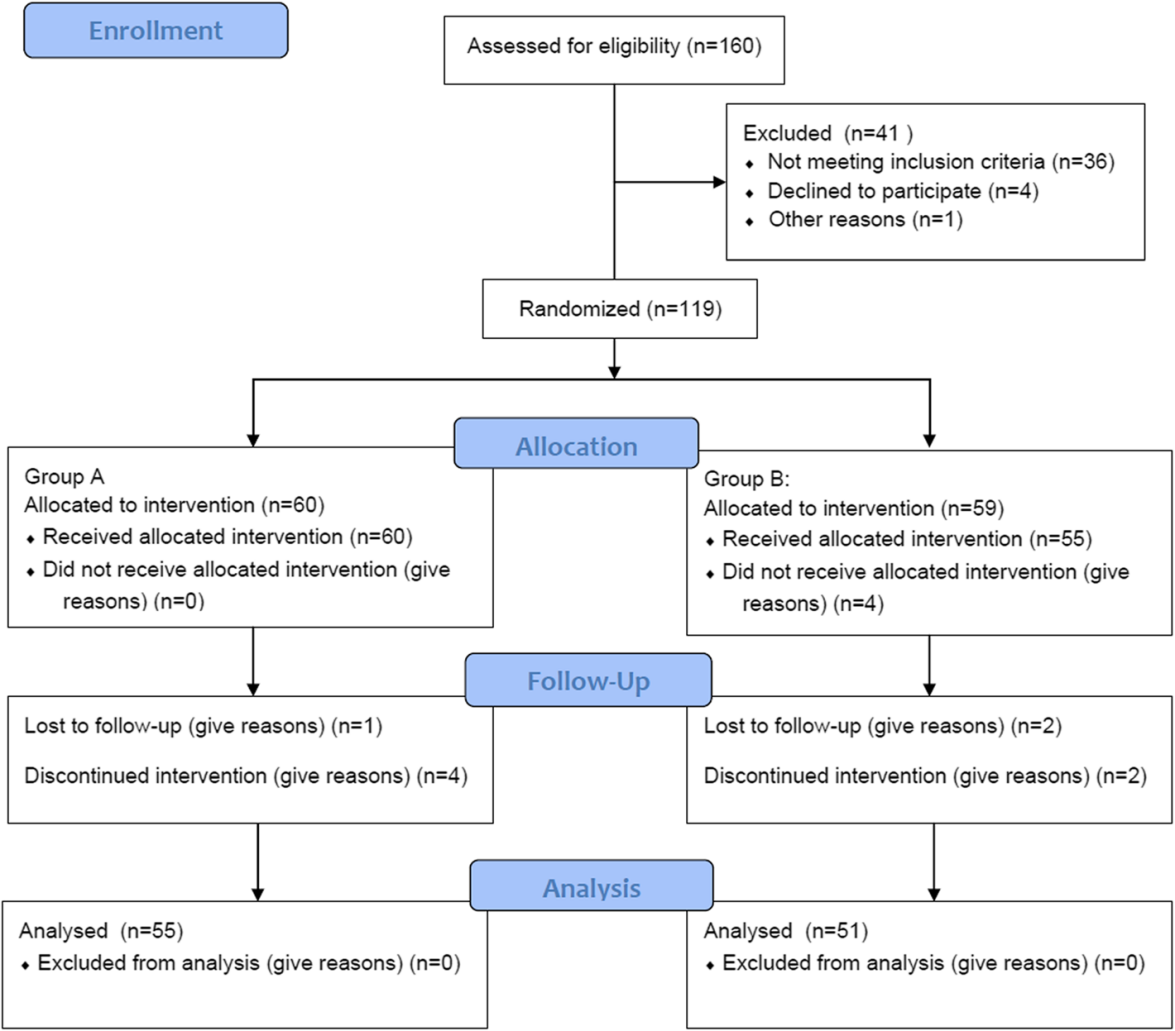
Flow diagram of this study

Forty-one patients were excluded due to ineligibility. Ultimately, 119 patients were randomly assigned to the SPT group (*n* = 60) or the KFEH group (*n* = 59) using a computer-generated randomized number table at a ratio of 1:1 (Fig. 1). After enrollment, demographic and knee joint function-related information was collected by an orthopedist.

### Rehabilitation program

Patients in the SPT group were instructed in accordance with a formal physical therapy program, and those in the KFEH group were educated about the home rehabilitation before TKA surgery, as described in Additional file 1. During the hospitalization period, all enrolled patients underwent the same functional exercise-based rehabilitation program with the aim of improving knee ROM, increasing knee and hip muscle strength, maintaining the length and elasticity of thigh tissues, preventing thrombosis, and acquiring the most important functional (i.e., activities of daily living) strategies. After discharge from hospital, patients in the SPT group participated in a total of 24 sessions of a physiotherapy and rehabilitation program 2 days/week for the first 7 weeks, followed by 1 day/month for the remaining 10 months of the year. This program consists of knee joint ROM exercises and strengthening exercises (including quadriceps setting exercise, straight leg raising exercise, stationary cycling as previously described [12], training strength and length were individually designed) for the knee joint after 20 min of application of moist heat and 20 min of transcutaneous electrical nerve stimulation. Standardized enhanced knee flexion home-based exercises included arrangement of knee joint motion and restoration of knee and hip muscle power. This program requires at least 5 days per week, 20 min/day, for 7 weeks, and 2–3 days per week for at least 10 months. The home program consisted of quadriceps femoris sets, hamstring sets, ankle pumps, terminal knee extension with weight, straight leg raises with weight in the supine and side-lying positions, cycling, and prone, hip, and knee flexion-extension with weight in supine, knee flexion-extension with weight in prone, and in sitting, static stretching exercises for hamstrings and gastrosoleus muscles [5, 13], as well as a low stool-assisted knee joint bending exercise (Additional file 1). Doctors in the team will guide patients through phone calls or WeChat to correct patient actions, answer patient questions once a week. Besides, doctors are always on call if the patient has any questions at any time.

### Primary outcome

For knee joint function measurements, patients from both groups underwent evaluations according to the Knee Society Score (KSS), the Western Ontario and McMaster Universities Osteoarthritis Index (WOMAC) score, and ROM assessment at selected time points (preoperatively; 1 week; 1, 3, and 6 months; and 1 year after surgery) as previously reported [14]. The visual analog scale (VAS), a widely used pain scale, was used to evaluate pain with movement at different time points [15]. Patients from both groups were instructed in the use of all measurement scales after enrollment. To reduce subjective bias, all questionnaires were administered by different orthopedists.

### Statistical analysis

All data were analyzed using SPSS version 20.0 (IBM Corporation, Armonk, NY, USA). Data were checked for normal distribution and are expressed as mean ± SD. KSS, WOMAC, ROM, and VAS scores were compared using the unpaired Student’s *t* test. Differences in KSS, WOMAC, ROM, and VAS between the two groups at different time points after surgery were compared using two-way analysis of variance, followed by Bonferroni’s post-test; differences with *P* < 0.05 were considered to be statistically significant.

## Results

After the exclusion of 41 patients, 119 were eligible and agreed to participate in the present study. Sixty and 59 patients were randomly assigned to the KFEH and SPT groups, respectively. All enrolled patients underwent TKA of only one knee. Four patients in the SPT group did not receive the allocated intervention (i.e., rehabilitation program) due to inconvenience with travel distance to the hospital and/or rehabilitation costs. One patient in the KFEH group was withdrawn from the study because she sustained a fracture due to a fall 3 months postoperatively. Three patients in the KFEH group and two in the SPT group were withdrawn from analysis to treat other systemic diseases postoperatively, which required inpatient nursing. One patient in the KFEH group and two in the SPT group were lost to follow-up at 3 or 6 months postoperatively because they did not wish to continue being evaluated. Ultimately, 55 patients in the KFEH group and 51 in the SPT group were included in the final analysis (Fig. 1).

The mean (± SD) age of the patients in the KFEH (11 male, 44 female) and SPT (8 male, 43 female) groups was 66.38 ± 8.35 and 67.27 ± 6.87 years, respectively. Other patient characteristics, including body mass index, sex, and diagnosis, are summarized in Table 1. There were no statistically significant differences in patient characteristics between the two groups.

**Table 1:** Mean ± SD of patient characteristics for group KFEH and group SPT

Items	Group KFEH (***n*** = 55)	Group SPT (***n*** = 51)
Age, years	68.4 ± 8.4	67.3 ± 6.9
Sex, n		
Female	44	43
Male	11	8
Side, n		
Right	38	36
Left	17	15
BMI	21.2 ± 1.5	21.5 ± 1.6
Weight, kg	56.8 ± 5.9	57.0 ± 5.7
Height, cm	163.8 ± 5.2	162.8 ± 4.9
Hospital stay, days	18.27 ± 3.76	17.43 ± 3.73

**Fig. 2:**
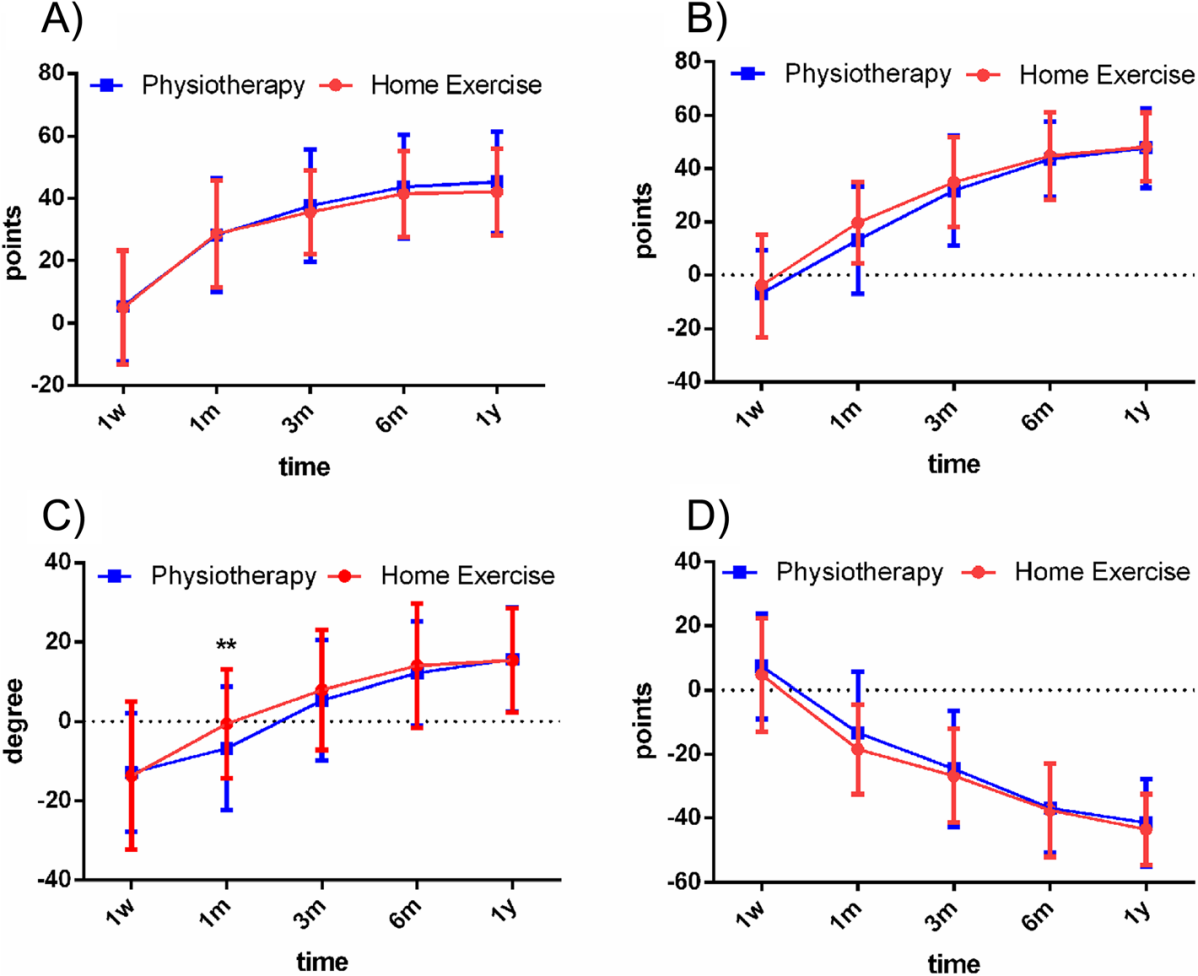
Differences of clinical outcomes between postoperative and preoperative. **A** KSS knee score; **B** KSS function score; **C** ROM; **D** WOMAC score. ** P < 0.01

Preoperative (Table 2) and postoperative (1 week; 1, 3, and 6 months; and 1 year) data were assessed for non-inferiority in each of the outcomes. Clinical outcomes at different periods during the 12-month follow-up period are summarized in Table 3. Pain and functional improvement(s) were observed in postoperative assessments in both groups during the 12-month follow-up. There was no statistical difference in VAS between the two groups at any time assessment. Overall ROM and functional scores, including KSS knee and function scores and WOMAC scores, were slightly better in the low stool-assisted home exercise program group (i.e., KFEH) at early follow-up. However, there were no statistical differences in these clinical outcomes between the KFEH and SPT groups during the 12-month follow-up, except for ROM at 1 month after surgery (*P* < 0.01) (Table 3). Comparison between preoperative and postoperative measures of each patient also revealed that the KFEH group experienced greater improvement in ROM at early follow-up (Fig. 2).

**Table 2:** Mean ± SD of preoperative data

Outcome	Group KFEH	Group SPT	***P***
KSS knee score, points	46.7 ± 12.0	44.2 ± 14.6	0.61
KSS function score, points	42.0 ± 11.6	44.0 ± 12.0	0.79
ROM, deg	100.0 ± 10.1	101.0 ± 10.2	0.58
VAS, points	4.3 ± 2.2	4.4 ± 2.2	0.83
WOMAC, points	52.7 ± 9.6	51.3 ± 11.4	0.18

*KSS* Knee Society Score, *ROM* range of motion, *VAS* visual analog scale, *WOMAC* Western Ontario and McMaster Universities Osteoarthritis Index

**Table 3:** Postoperative outcome by treatment group (mean ± SD)

Outcome	Group KFEH	Group SPT	***P***
KSS knee score, points			
Wk 1	51.7 ± 14.9	49.7 ± 12.0	0.45
Mo 1	75.3 ± 9.6	72.5 ± 11.0	0.16
Mo 3	82.3 ± 7.3	81.9 ± 8.6	0.78
Mo 6	88.3 ± 7.2	87.9 ± 7.4	0.81
Yr 1	88.8 ± 7.8	89.5 ± 7.5	0.64
KSS function score, points			
Wk 1	38.2 ± 15.0	37.2 ± 12.1	0.70
Mo 1	61.6 ± 11.3	57.2 ± 15.0	0.09
Mo 3	76.8 ± 13.1	75.8 ± 14.0	0.70
Mo 6	86.7 ± 11.1	87.6 ± 106	0.70
Yr 1	90.2 ± 10.1	91.8 ± 9.2	0.40
ROM, deg			
Wk 1	86.2 ± 12.9	88.2 ± 10.3	0.40
Mo 1	99.4 ± 8.5	94.2 ± 9.8	< 0.01
Mo 3	107.9 ± 10.5	106.4 ± 11.2	0.48
Mo 6	114.0 ± 10.4	113.2 ± 7.9	0.66
Yr 1	115.3 ± 8.2	116.7 ± 8.9	0.42
VAS, points			
Wk 1	5.2 ± 1.7	5.5 ± 1.2	0.42
Mo 1	2.6 ± 1.1	2.7 ± 1.3	0.49
Mo 3	1.7 ± 1.0	1.6 ± 1.1	0.90
Mo 6	1.1 ± 1.0	1.0 ± 0.9	0.61
Yr 1	0.9 ± 0.8	0.8 ± 0.8	0.75
WOMAC, points			
Wk 1	57.5 ± 14.4	58.7 ± 11.5	0.63
Mo 1	34.2 ± 10.6	38.0 ± 13.8	0.12
Mo 3	25.9 ± 11.7	16.7 ± 12.4	0.76
Mo 6	15.2 ± 10.0	14.4 ± 9.6	0.67
Yr 1	9.2 ± 9.1	9.9 ± 8.2	0.68

*SD* standard deviation, *deg* degrees, *Wk* week, *Mo* month, *Yr* year, *KSS* Knee Society Score, *ROM* range of motion, *VAS* visual analog scale, *WOMAC* Western Ontario and McMaster Universities Osteoarthritis Index

An analysis of the average total cost of the first 2 months of supervised physiotherapy and the home-based knee flexion exercise program was performed. The approximate total costs were 1805 RMB in the SPT group and 1023 RMB in KFEH group. Patients in the KFEH group could save approximately 800 RMB after 2 months of rehabilitation (details summarized in Table 4).

**Table 4:** Cost analysis of group KFEH and group SPT (monetary RMB)

Applications	Group KFEH	Group SPT
**Assessment price**	**140 (20 × 7)**	**140 (20 × 7)**
Preoperative assessment	20	20
Outpatients clinic assessment	20	20
Assessment at 2 weeks	20	20
Assessment at 1 month	20	20
Assessment at 3 months	20	20
Assessment at 6 months	20	20
Assessment at 12 months	20	20
**Physiotherapy items**	**105**	**1505 (100 × 14 + 105)**
Weekly postoperative guide	105 (15 × 7)	105 (15 × 7)
Warm heat application	0	10
ROM and strength exercises	0	45
TENS	0	45
**Exercise equipment**	**730**	**0**
Low stool	30	0
Stationary bicycle	700	0
**Transportation fee (round trip)**	**48 (8 × 6)**	**160 (8 × 20)**
**Total cost**	1023	1805

Regarding complications, there was no DVT, infection, or tendon tears during exercise in either group. However, one patient in the KFEH group sustained a fracture in a fall at 3 months and was followed-up by hospital care and, therefore, was excluded from data analysis.

## Discussion

After 12 months of follow-up assessment, our study observed good outcomes in the first month in patients who underwent the home-based enhanced knee flexion exercise program (i.e., KFEH). The KFEH group demonstrated better ROM range at 1 month than the SPT group (*P* < 0.01). No inferiority was revealed regarding WOMAC, KSS, and VAS scores between the two groups, as well as the complication rate. Not surprisingly, results of the present study revealed that the home-based program could also lower the burden on patients’ time and costs related to in-hospital rehabilitation.

With aging societies in many parts of the world, the number of patients undergoing TKA surgery has increased globally in recent years [16, 17]. In addition to excellent surgeon skills, postoperative rehabilitation is considered to have a significant effect on patients’ knee function and satisfaction with surgery [18, 19]. Due to the high costs of physiotherapy, an effective home-based program protocol needs to be implemented and expanded in China. By chance, we observed that a group of patients who exercised a habit of sitting on small low stools achieved faster rehabilitation and better satisfaction after TKA surgery. We hypothesized that sitting on a low stool could improve ROM, especially knee flexion, and help patients achieve faster and better rehabilitation after TKA. Some studies have demonstrated that increasing ROM is important for patient functional outcomes and satisfaction after TKA [20, 21].

Knee ROM is an objective variable used to evaluate final flexion after TKA. With a postoperative ROM of between 100 and 120°, most activities of daily life can be performed comfortably [22, 23]. One challenge of home-based rehabilitation is the possibility of unsatisfactory knee flexion rehabilitation out-of-hospital due to unclear recovery goals and poor exercise habits. The advantage of the KFEH program is to establish a proper target and self-administered rehabilitation test when we asked patients in the KFEH group to perform flexion practice while sitting on a low stool. In our study, ROM demonstrated an increasing trend after surgery in both groups. There was a clear difference in the first month, which showed that patients in the home-based exercise program involving low stool assistance (i.e., KFEH) exhibited larger mean knee joint ROM than those in the SPT group (99.4 ± 8.5° versus 94.2 ± 9.8°, respectively; *P* < 0.01). The change in pre- and postoperative ROM also had similar results, as well as the absolute values of the KSS pain and function scores and the WOMAC scores, which suggest that patient satisfaction was somewhat higher in the KFEH group. These outcomes suggest that use of a low stool may facilitate improvement in knee joint ROM after TKA.

The KSS is a clinical rating system published in 1989 to measure the knee in patients undergoing TKA [24]. WOMAC was developed to evaluate pain, stiffness, and functional limitation of patients with OA by Bellamy in 1982 [25]. In our study, there were no significant differences between the KFEH and SPT groups with regard to KSS or WOMAC results, although absolute values of the KSS and the WOMAC scores were better in the KFEH group during the first 3 months after surgery. Several factors contributing to knee function or satisfaction and outcomes vary from patient to patient. Some studies have demonstrated that patient perceptions of function could differ from actual function, and patient factors, including obesity, motivation, and fatigue, could also affect the results [14, 21, 26]. This may explain the differences in ROM, an individual factor change, is unlikely to completely influence the KSS or WOMAC results.

The costs and inconvenience associated with physiotherapy are sources of concern for patients who undergo SPT after TKA, which usually includes the application of heat, ROM exercises, strength recovery, and other applications to avoid postoperative conditions such as loss of motion in the joint, muscle atrophy, tissue edema, and functional limitations [27]. It is recommended that patients undergo physiotherapy training two or three times weekly to achieve these goals [28]. A study from the USA demonstrated that Medicare reimbursements for physical therapy would be > $1000 for 12 sessions; as such, home-based rehabilitation could significantly lower the economic burden on patients [14]. In our study, the home-based exercise program involved several actions to rehabilitate ROM of the joint, muscle strength, and gait balance. Small low stools can help patients enhance knee joint flexion during exercise. The total cost for each group mainly consists of assessments and training applications. The approximate total cost of 2 months’ rehabilitation were 1805 RMB in the physiotherapy group and 1023 RMB in the home exercise group according to a crude analysis. Considering that the resident income in China in 2016 was 23,821 RMB [29], the home-based exercise program would lower the economic burden on patients, taking into account the undervalued work of medical staff in China.

In addition to functional recovery, we also investigated whether the act of sitting on a low stool would cause ligament injury during rehabilitation. After 12 months’ follow-up, there was no record of ligament tears or severe pain around the knee joint nor was there any evidence of DVT, infection, or other complications. Importantly, the safety of the low stool-assisted home-based exercise program is considered to be non-inferior compared with supervised physiotherapy.

This study had some limitations, the first of which was that the assessor physiotherapist was not blinded to patient allocation. Second, patient compliance in the KFEH group was good because of regular follow-up and scoring; however, there is no guarantee that we could obtain the same results from patients with only fair or poorer compliance, such as those who refused to participate in the study, or those who become weary or annoyed with long-term follow-up. Third, physical therapists differed for each patient, as well as the details of the therapy protocol and frequency, which would inevitably result in individual differences. Fourth, the socioeconomic and educational status of participants were not collected and analyzed between the two groups. Finally, the sample size was insufficient to evaluate some outcome differences between the two groups; therefore, studies involving more participants are warranted.

## Conclusion

In conclusion, findings of the present study suggest that the self-developed home-based enhanced knee flexion exercise program resulted in better ROM at early discharge. VAS, KSS, and WOMAC scores, and complication rates during the 12-month follow-up period were non-inferior to SPT. Furthermore, we verified the efficiency and cost-effectiveness of using a home-based program for TKA rehabilitation. This home-based program might reduce the risk of coronavirus infection because of the minimizing face to face contact.

## Supplementary Information

**Additional file 1.** The Guide of Low Stool Assisted Home Exercise after Total Knee Arthroplasty.

## Data Availability

All data generated or analyzed during this study are included in this published article.

## Abbreviations

SPT: Supervised physiotherapy
KFEH: Knee flexion exercise program involving a low stool
TKA: Total knee arthroplasty
KSS: Knee Society Score
VAS: Visual analog scale
WOMAC: Western Ontario and McMaster Universities Osteoarthritis Index
ROM: Range of motion
OA: Osteoarthritis
