# Supplementary material for: Efficacy and safety of a self-developed home-based enhanced knee flexion exercise program compared with standard supervised physiotherapy to improve mobility and quality of life after total knee arthroplasty: a randomized control study
Source: J Orthop Surg Res. 2021 Jun 14;16:382. doi: 10.1186/s13018-021-02516-0 (PMC8202539; doi:10.1186/s13018-021-02516-0)
Supplement: Supplementary file 1 — Additional file 1. The Guide of Low Stool Assisted Home Exercise after Total Knee Arthroplasty. [file 13018_2021_2516_MOESM1_ESM.doc]

**Additional file 1. The Guide of Low Stool Assisted Home Exercise after Total Knee Arthroplasty**

**Part 1. Extension Practice**


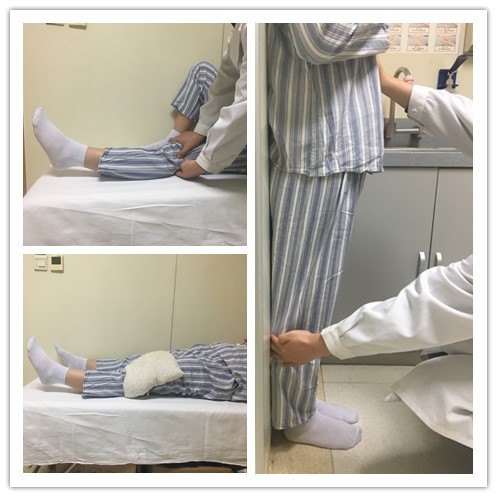


**Figure 1.**

Requirement: No fingers should be inserted into the space between the wall and shank when standing against the wall with fully straight knee joint.


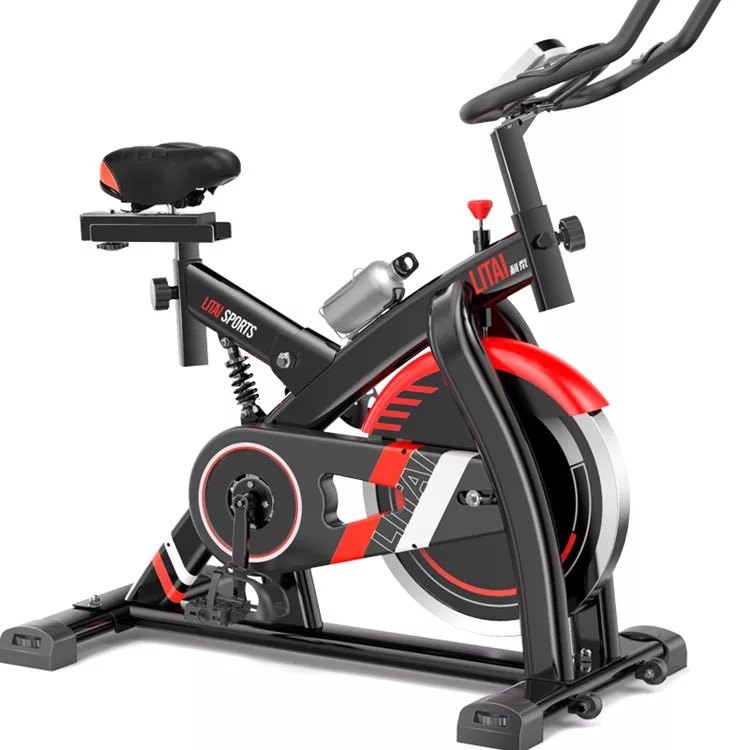


**Figure 2.**

Exercycling is an excellent activity and helpful to regain muscle strength and knee mobility. Before exercycling training, adjust the seat height so that the bottom of your foot just touches the pedal with your knee almost straight and bend.


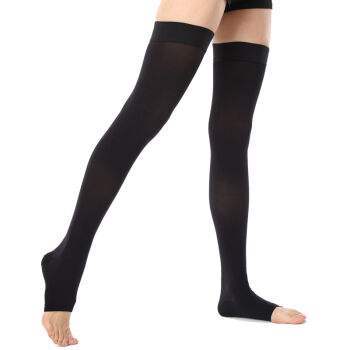


**Figure 3. Elastic socks**

Recommendation: Elastic socks are helpful to prevent thrombosis, enhance muscle strength and protect the knee joint. (3-8 weeks elastic socks wearing after surgery).

**Part 2. Flexion Practice**


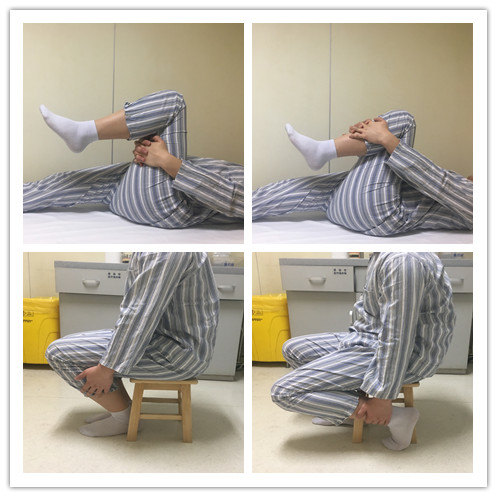


**Figure 4. Flexion practice：**

Requirement: Practice to bend the knee into a comfortable position as far as possible in a step-by-step approach. You can use the low stool (30cm-40cm in height) assisting flexion practice as the photos show. It is better to bend the knee as far as possible though it is inevitably painful, or it will lead to daily difficulties such as in stair climbing.

**Part 3. Strength training**


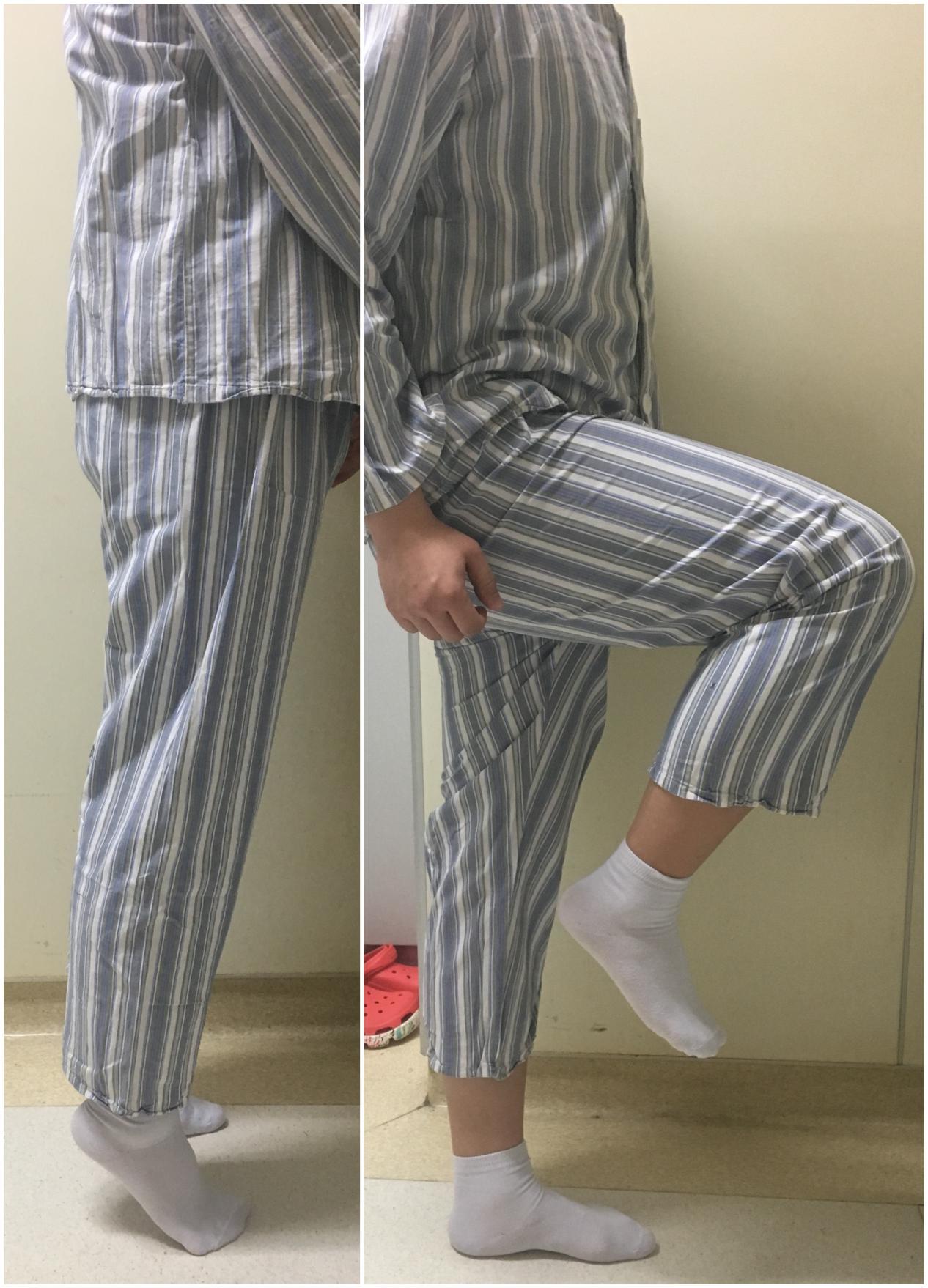


**Figure 5. Strength training：**

Requirement: 1. Stand on the tip toes as higher as possible and hold the position for 5-10 seconds before recovering for relaxing, this strength training should be repeated at a interval of 5-10 seconds.

2. Practice to stand with the affected leg, with holding the wall for support and balance with the ipsilateral hand to avoid falling.


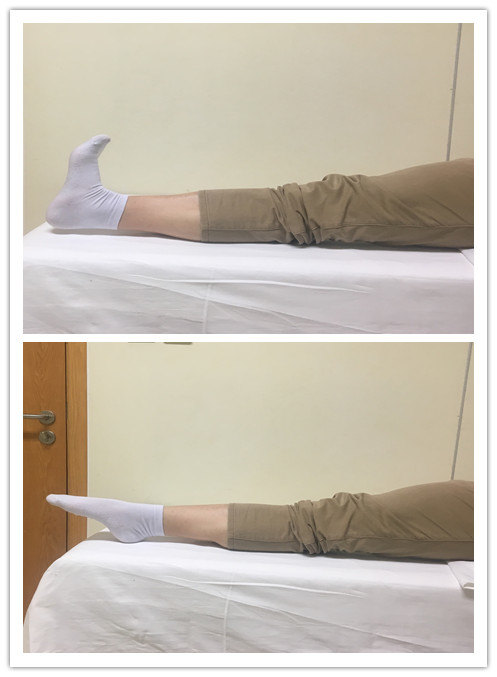


**Figure 6.**

Move the foot up to bend the ankle joint by contracting the calf and hold for 5 seconds, and then move the foot down as far as possible and hold for another 5 seconds. This performance will be helpful to promote blood circulation, reduce swelling, avoid thrombosis and enhance quadriceps strength. and shin muscles.


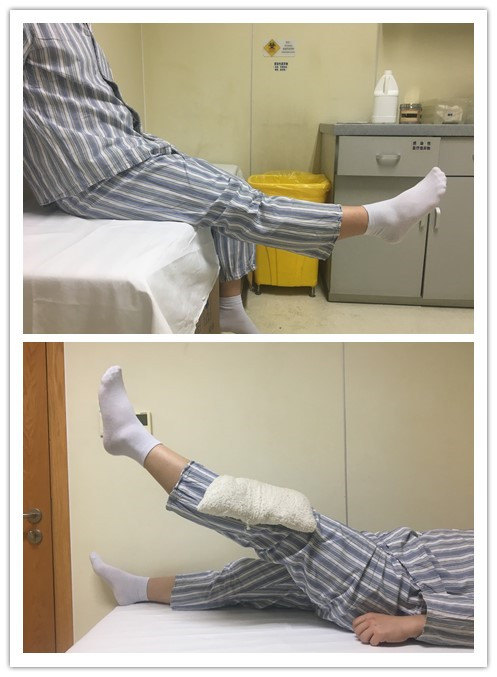


**Figure 7.**

Sit on the edge of bed, lift the affected leg to hold the knee fully straightened with the leg unsupported.

Lie on the bed, lift the affected leg to 45 degree with a 5-7 kg of load on the knee.

Guidelines

1. You should be doing about 8 -15 repetitions of each exercise. The exercise usually takes 20-40 minutes per day.

2. Train 5 days per week at first 8 weeks.

3. Train 2-3 days per week to maintain current condition after 2 months practice.

4. Ask surgeons or physiotherapists if you have any problems or discomforts.

5. Remember assess your achievements during follow up.
